# EZH2 immunoexpression in pleomorphic adenoma and adenoid cystic carcinoma and clinicopathological features

**DOI:** 10.1590/1807-3107bor-2024.vol38.0018

**Published:** 2024-03-11

**Authors:** Mariana Saturnino de NORONHA, Karolina Skarlet Silva VIANA, Maria Cássia Ferreira de AGUIAR, Cristiane Helena SQUARIZE, Mauro Henrique Nogueira Guimarães de ABREU, Elismauro Francisco MENDONÇA, Vanessa de Fátima BERNARDES

**Affiliations:** (a) Universidade Federal de Minas Gerais – UFMG, School of Dentistry, Department of Oral Surgery and Pathology, Belo Horizonte, MG, Brazil.; (b) University of Michigan, School of Dentistry, Department of Periodontics and Oral Medicine, Ann Arbor, MI, USA.; (c) Universidade Federal de Minas Gerais – UFMG, School of Dentistry, Department of Community and Preventive Dentistry, Belo Horizonte, MG, Brazil.; (d) Universidade Federal de Goiás – UFG, School of Dentistry, Department of Stomatologic Sciences, Goiânia, GO, Brazil.; (e) Universidade Federal de Minas Gerais – UFMG, Biological Sciences Institute, Department of Pathology, Belo Horizonte, MG, Brazil.

**Keywords:** Immunohistochemistry, Enhancer of Zeste Homolog 2 Protein, Salivary Gland Neoplasms, Carcinoma, Adenoid Cystic, Adenoma, Pleomorphic

## Abstract

The aim of this study was to evaluate the expression of the EZH2 protein and describe the clinical and microscopic characteristics of adenoid cystic carcinoma (ACC) and pleomorphic adenoma (PA). The study included 16 ACC cases and 12 PA. All ACC and PA cases were positive for EZH2 and the ACC samples showed significantly higher EZH2 expression. The clinical and microscopic covariates were described in relation to EZH2 staining in ACC samples. The highest mean values of EZH2 were observed in cases with local metastasis, recurrence, perineural invasion, and predominantly cribriform growth pattern without solid areas. EZH2 is a potential marker of malignancy.

## Introduction

Although adenoid cystic carcinoma (ACC) and pleomorphic adenoma (PA) originate from the same segment of the salivary gland, the intercalated duct, these tumors differ in their nature and clinical behavior. ACC is an aggressive tumor that frequently presents with distant metastasis to the lungs, and the overall survival rate at 15 years is approximately 25%.^1,2^ PA is a benign salivary gland neoplasia characterized by slow growth and eventual recurrence. Both tumors can benefit from target therapy associated with genetic and epigenetic alterations.^3^

**Figure 1:**
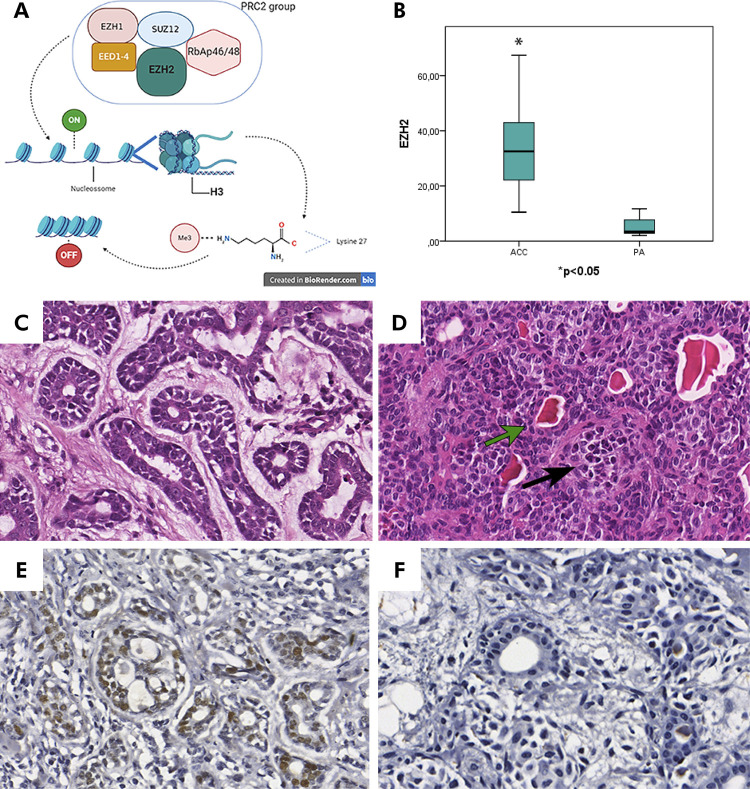
A: EZH2 is a core component of PRC2 and is responsible for trimethylation of the H3 Lysine 27. This role is crucial to the chromatin organization around histones altering gene expression. B: Significant difference between ACC and AP EZH2 protein expression (Mann Whitney U). C: Adenoid Cystic Carcinoma (Hematoxylin and Eosin), shows myoepithelial and glandular epithelial cells arranged as duct-like structures (400x). D: Pleomorphic adenoma (Hematoxylin and Eosin) shows glandular epithelial cells and myoepithelial cells. Green arrow highlights duct-like structures and black arrow highlights myoepithelial cells in a plasmacytoid shape (400x). E: EZH2 IHC on the Adenoid Cystic Carcinoma showing diffuse nuclear staining in neoplastic cells (400x). F: EZH2 IHC of Pleomorphic adenoma shows glandular epithelial cells, myoepithelial cells and duct-like structures (400X).

The enhancer of zeste homolog 2 protein (EZH2) contributes to epigenetic alterations, specifically histone modifications. EZH2 is a core enzymatic component of polycomb repressive complex 2 (Figure 1A), associated with rapid progression in various cancers such as prostate and breast cancer.^4,5^ Information regarding the association of the ACC histopathological patterns and clinical characteristics with the EZH2 protein expression is scarce. Here, we present results showing the immunohistochemical (IHC) expression of the EZH2 protein in ACC and PA aiming to evaluate the expression of the EZH2 protein and to describe the clinical and histopathologic features of ACC and PA.

## Methodology

This study was approved by the Institutional Research Ethics Committee (12235719.9.0000.5149). Paraffin blocks were obtained from the archives of the Oral and Maxillofacial Pathology Service of the Federal University of Minas Gerais (Belo Horizonte, MG, Brazil).

All hematoxylin and eosin stained slides were reviewed by two experienced oral pathologists (MCFA and VFB) who performed a qualitative analysis. The inclusion criteria for ACC and PA followed the WHO classification.^1^

The calculation of power was made a posteriori, rejecting the null hypothesis with a probability of 100%. The measures of central tendency and variability for EZH2 were described for the studied covariates. IHC was performed using a two-step method. The slides were incubated with anti-EZH2 monoclonal antibody (1:50, D2C9, Cell Signaling, Danvers, MA, USA) for 1 hour at room temperature and 18 hours at 4°C. Slides were then treated with EnVision+ Dual Link System-HRP (Dako, Carpinteria, USA), and 3.3’-Diaminobenzidine was used as the chromogen (Dako, Carpinteria, USA).

Positive controls consisted of tonsil samples, and negative controls were carried out by omitting the primary antibody. IHC positivity was evaluated in 10 high-power fields (400× magnification) by a trained examiner. EZH2 was expressed in the nucleus, and positive and negative tumor cells were counted. The average percentage of positive cells was calculated. Statistical analysis was performed using SPSS software (IBM SPSS Statistics for Windows, Version 20.0. Armonk, USA).

## Results

The study included 16 cases of ACC and 12 cases of PA. The mean age of ACC patients was 53.13 years and the male-to-female ratio was 1.66 (Table 1). In PA cases, the mean age was 44.5 years and male-to-female ratio was 0.5 (Table 2). The location of the PA cases was palate (9), vestibule (1), and buccal mucosa (2) while the location of the ACC cases was palate (3), buccal mucosa (1), parotid gland (4), submandibular gland (4), sublingual gland (3), and tongue (1). The histopathological analysis of ACC samples showed the three growth patterns: tubular, cribriform, and solid forms (Figure 1C). PA showed myoepithelial and glandular epithelial cells arranged as duct-like structures and sheets intermingled in the variable stroma (Figure 1D).

**Table 1:** Analysis and measures of central tendency and variability of EZH2 expression in ACC samples.

Covariates	Absolute number (n)	ACC samples (%)	Mean (SD) (%)	Minimum (%)	Median (%)	Maximum (%)
Growth pattern						
Cribriform	8	50.0	37.87 (17.20)	19.12	36.67	67.38
Tubular	6	37.5	27.78 (13.69)	10.45	32.78	43.20
Solid	2	12.5	29.73 (5.68)	25.71	29.73	33.75
Solid areas						
Yes	4	25.0	25.44 (6.16)	19.12	24.44	43.20
No	12	75.0	35.61 (16.48	10.45	35.27	67.38
Perineural invasion						
Yes	7	43.7	33.79 (10.23)	21.00	31.26	50.98
No	9	56.3	32.50 (18.64)	10.45	34.31	67.38
Nodal metastasis						
Yes	6	37.5	30.97 (7.43)	25.71	30.97	36.23
No	10	62.5	27.43 (13.03)	10.45	26.93	50.98
Recurrence*						
Yes	11	15.0	36.96 (8.05)	31.26	36.96	42.66
No	2	85.0	28.21 (13.34)	10.45	25.71	50.98

*For recurrence 3 samples did not have the data available.

**Table 2:** Features and EZH2 expression in PA samples.

Sample	Sex	Age	Site	EZH2 index expression
1	F	32	Palate	8.59
2	F	39	Palate	11.70
3	F	70	Palate	3.16
4	F	45	Palate	2.91
5	M	56	Palate	3.17
6	M	13	Palate	3.78
7	F	37	Palate	2.01
8	M	49	Vestibule	2.18
9	F	53	Palate	2.75
10	M	53	Palate	3.59
11	F	35	Buccal mucosa	6.68
12	F	52	Buccal mucosa	8.57

All ACC and PA cases were positive for EZH2, with diffuse nuclear staining in neoplastic cells (Figure 1E and 1F). Comparatively, ACC samples showed more positive neoplastic cells than PA samples (Figure 1B, *p < 0.05). After a stratified analysis by sex, age, and location (large or minor salivary gland), the difference remained significant. No strong positivity was observed in the adjacent normal salivary gland.

In ACC cases, we described the EZH2 marker based on clinical and histopathological aspects, such as the predominant growth pattern and the presence or absence of solid areas, perineural invasion, nodal metastasis, and recurrence. The measures of central tendency and variability are shown on Table I. Notably, the highest mean values of EZH2 were observed in cases with local metastasis, recurrence, and a predominantly cribriform growth pattern without solid areas and perineural invasion. The findings suggest that advanced clinical stage, represented by recurrence and nodal metastasis, is more associated with high levels of EZH2 than the histopathological pattern, giving a new prognostic value for EZH2.

## Discussion

Despite the limited sample size, EZH2 emerged as a reliable biomarker for malignancy of salivary gland tumors. The present study showed positive staining in PA and ACC samples, but with a remarkable quantitative difference. We found low expression of EZH2 in PA, which is consistent with a previous study showing negative expression of EZH2 in benign salivary gland tumors.^6^ Thus, EZH2 seems to be relevant for differentiating malignant from benign salivary gland tumors. Similar results were found in other studies of glandular neoplasms, such as breast and prostate cancer.^4,5,7^

EZH2 is an independent biomarker of breast cancer risk in normal breast samples, helping to determine the malignancy risk in benign biopsies.^4^ The investigation of such possibility in benign salivary tumors deserves attention.

It is well known that the solid variant of ACC and perineural invasion are associated with poor prognosis,^7^ and we observed a slightly higher index of EZH2 in cases with perineural invasion. We found that cases with recurrence and nodal metastasis presented a higher EZH2 mean value, suggesting the prognostic value of EZH2 for ACC cases.

EZH2 is involved in epithelial-to-mesenchymal transition by interacting with Snail1 and downregulating the expression of E-cadherin.^8^ As a result, EZH2 may increase metastasis. Metastasis to the lung is a major feature of ACC. Therefore, further studies are essential to elucidate the role of EZH2 in the progression and distant metastasis of ACC.

## Conclusions

EZH2 overexpression in ACC has the potential to be a biomarker of malignancy and advanced progression. In benign salivary gland tumors, the role of EZH2 as a tool to show malignant transformation deserves investigation. It is difficult to establish a diagnosis and prognosis of salivary gland tumors. Since there are similarities between malignant and benign tumors, our goal was to validate the auxiliary parameters for prognosis of ACC and PA. Despite the fact that ACC and PA share a well-established morphological feature, a detailed study of EZH2 protein expression according to clinical and histopathological aspects can improve our understanding of ACC behavior.
